# Supplementary material for: Predictors of life-threatening complications in relatively lower-risk patients hospitalized with COVID-19
Source: PLoS One. 2022 Feb 15;17(2):e0263995. doi: 10.1371/journal.pone.0263995 (PMC8846540; doi:10.1371/journal.pone.0263995)
Supplement: S1 Table — (DOCX) [file pone.0263995.s001.docx]

| **S1 Table. List of comorbid conditions and associated ICD-10 diagnoses codes** | |
| --- | --- |
| **Comorbidity** | **ICD10 Code** |
| chronic ischemic heart disease | I21 – Acute myocardial infarction* |
|  | I22 – ST elevation myocardial infarction, non-ST elevation myocardial infarction* |
|  | I25 – Chronic ischemic heart disease |
| congestive heart failure | I09.9 – Rheumatic heart disease, other |
|  | I11.0 – hypertensive heart disease with heart failure |
|  | I13.0 – hypertensive heart and chronic kidney disease |
|  | I42.0 – dilated cardiomyopathy |
|  | I42.5-I42.9 – other cardiomyopathies |
|  | I43 – cardiomyopathy in diseases classified elsewhere |
|  | I50 – heart failure |
|  | P29.0 – neonatal cardiac failure |
| peripheral vascular disease | I70 – atherosclerosis |
|  | 171 – aortic aneurysm and dissection |
|  | I73.1 – thromboangiitis obliterans |
|  | I73.8 – other specified PVD |
|  | I73.9 – PVD, unspecified |
|  | I77.1 – stricture of artery |
|  | I79.0 – aneurysm of aorta in diseases classified elsewhere |
|  | K55.1 – chronic vascular disorders of intestine |
|  | K55.8 – other vascular disorders of intestine |
|  | K55.9 - vascular disorders of intestine, unspecified |
|  | Z95.8 – presence of other cardiac and vascular implants and grafts |
|  | Z95.9 – presence of cardiac and vascular implants and graft, unspecified |
| cerebrovascular disease | G45 – transient cerebral ischemic attacks and related syndromes |
|  | G46 – vascular syndromes of brain in cerebrovascular diseases |
|  | H34 –retinal artery occlusions |
|  | I60 – nontraumatic subarachnoid hemorrhage |
|  | I61 – nontraumatic intracerebral hemorrhage |
|  | I62 – other and unspecified nontraumatic intracranial hemorrhage |
|  | I63 – cerebral infarction* |
|  | I65 – Occlusion and stenosis of precerebral arteries, no resulting in cerebral infarction |
|  | I66 – Occlusion and stenosis of cerebral arteries, no resulting in cerebral infarction |
|  | I67 –Other cerebrovascular disease |
|  | I68 – Cerebrovascular disorders in diseases classified elsewhere |
|  | I69 – Sequelae of cerebrovascular disease |
| chronic pulmonary disease | I27.8 – Other specified pulmonary diseases (Chronic) |
|  | I27.9 – pulmonary heart disease, unspecified |
|  | J40-J47 – chronic lower respiratory diseases: bronchitis, not specified as acute or chronic; simple and mucopurulent chronic bronchitis; unspecified chronic bronchitis; emphysema; other chronic obstructive pulmonary disease; asthma; bronchiectasis |
|  | J60-J67 – lung diseases due to external agents: coal worker’s pneumoconiosis, pneumoconiosis due to asbestos, dust containing silica, other inorganic dusts, unspecified; pneumoconiosis associated with tuberculosis; airway disease due to specific organic dust; hypersensitivity pneumonitis due to organic dust |
|  | J68.4 – chronic respiratory conditions due to chemical, gases, fumes and vapors |
|  | J70.1 – chronic and other pulmonary manifestations due to radiation |
|  | J70.3 – chronic drug-induced interstitial lung disease |
| dementia | F01 – vascular dementia |
|  | F02 – dementia in other diseases classified elsewhere |
|  | F03 – unspecified dementia |
|  | F05.1 – delirium superimposed on dementia |
|  | G30 – Alzheimer’s disease |
|  | G31 – other degenerative disease so nervous system, not otherwise classified |
|  | R54 – age-related physical debility |
| connective tissue disease | M05-M06 – rheumatoid arthritis, other rheumatoid arthritis |
|  | M31.5 – giant cell arteritis with polymyalgia rheumatica |
|  | M32-M34 – systemic lupus erythematosus, dermatopolymyositis, systemic sclerosis |
|  | M35.1 – other overlap syndromes |
|  | M35.3 – polymyalgia rheumatica |
|  | M36.0 – dermatopolymyositis in neoplastic disease |
| peptic ulcer disease | K25-K28 – gastric ulcer, duodenal ulcer, peptic ulcer, site unspecified, gastrojejunal ulcer |
| liver disease | K70 – alcoholic liver disease |
|  | K71.3-71.5 – toxic liver disease with chronic hepatitis |
|  | K71.7 – toxic liver disease with fibrosis and cirrhosis of liver |
|  | K72.1 – chronic hepatic failure |
|  | K72.9 – hepatic failure, unspecified |
|  | K73-K74 – chronic hepatitis, not elsewhere classified; fibrosis and cirrhosis of liver |
|  | K76 – other diseases of liver: fatty (change of) liver, not elsewhere classified; chronic passive congestion; central hemorrhagic necrosis; infarction of liver; peliosis hepatis; hepatic veno-occlusive disease; portal hypertension; hepatorenal syndrome; other specified diseases of liver, hepatopulmonary syndrome; liver disease, unspecified |
| diabetes mellitus | E10-E14 – Type 1 and Type 2 Diabetes, and unspecified |
| hemiplegia | G04.1 – tropical spastic paraplegia |
|  | G11.4 – hereditary spastic paraplegia |
|  | G81 –hemiplegia and hemiparesis |
|  | G82 – paraplegia and quadriplegia |
|  | G83.0-G83.4 – other paralytic syndromes |
|  | G83.9 – paralytic syndrome unspecified |
| moderate to severe chronic kidney disease | I12.0 – hypertensive chronic kidney disease with stage 5 CKD or ESRD |
|  | I13.1 – hypertensive heart disease and CKD w/o HF |
|  | N03.2-N03.7 – chronic nephritic syndromes |
|  | N05.2-N05.7 – unspecified nephritic syndromes |
|  | N18-N19 – CKD and unspecified kidney failure |
|  | N25.0 – renal osteodystrophy |
|  | Z49.0-Z49.2 – encounters for care involving renal dialysis |
|  | Z94.0 – kidney transplant status |
|  | Z99.2 – dependence on renal dialysis |
| solid tumor, leukemia, lymphoma | C00-C14 – malignant neoplasms of lip, oral cavity and pharynx |
|  | C15-C26 – malignant neoplasms of digestive organs |
|  | C30-C34 – malignant neoplasms of respiratory |
|  | C37-C41 – malignant neoplasms of intrathoracic organs and bone and articular cartilage |
|  | C43 – malignant melanoma of skin |
|  | C45-C58 – malignant neoplasms of mesothelial and soft tissue, breast, female genital organs |
|  | C60-C85, C88, C90-97 – malignant neoplasms of male organ, urinary tract, eye brain and CNS, thyroid and other endocrine, ill defined, lymphoid, hematopoietic and related tissue |
| AIDS | B20-B22 – HIV resulting in infections, malignant neoplasms, or other unspecified diseases |
|  | B24 – unspecified HIV disease |
| *ICD codes utilized only if coded prior to the date of hospitalization | |
