# Supplementary material for: Predictors of life-threatening complications in relatively lower-risk patients hospitalized with COVID-19
Source: PLoS One. 2022 Feb 15;17(2):e0263995. doi: 10.1371/journal.pone.0263995 (PMC8846540; doi:10.1371/journal.pone.0263995)
Supplement: S2 Table — (DOCX) [file pone.0263995.s002.docx]

| **S2 Table. Clinical outcomes for subgroups of relatively lower-risk patients admitted with**  **COVID-19** | | | | | | | |
| --- | --- | --- | --- | --- | --- | --- | --- |
|  |  | Relatively lower-risk Sample, Without Hypertension, Obesity*, Pregnancy or Smoking History | | | | Relatively lower-risk Sample, less than 35 years old | |
|  |  | <55 years old | | ≥55 years old | | <35 years old | |
| Total patients, n | | 235 |  | 244 |  | 104 |  |
| Any new life-threatening complication†, n (%) | | 29 | 12.3% | 88 | 36.1% | 9 | 8.7% |
|  | Septic shock | 8 | 3.4% | 20 | 8.2% | 1 | 1.0% |
|  | Positive blood culture | 8 | 3.4% | 13 | 5.3% | 4 | 3.9% |
|  | Renal replacement therapy | 4 | 1.7% | 11 | 4.5% | 0 | 0 |
|  | Major arrhythmias | 6 | 2.55% | 12 | 4.9% | 0 | 0 |
|  | New myocardial infarction | 1 | 0.4% | 3 | 1.2% | 0 | 0 |
|  | Heart failure or cardiogenic shock | 1 | 0.4% | 4 | 1.6% | 0 | 0 |
|  | Confirmed venous thrombus embolism | 2 | 0.9% | 14 | 5.7% | 0 | 0 |
|  | Disseminated intravascular coagulation | 1 | 0.4% | 4 | 1.6% | 0 | 0 |
| Intubated, n (%) | | 19 | 8.1% | 50 | 20.5% | 6 | 5.8% |
|  | Time-to-intubation in days, mean (SE) | 5.6 | 6.7 | 4.5 | 3.9 | 3.3 | 4.9 |
| Death, n (%) | | 11 | 4.7% | 61 | 25.0% | 2 | 2% |
|  | Time-to-death in days, mean (SE) | 20.8 | 18.4 | 10.8 | 8.1 | 12.5 | 16.3 |
| Length of hospitalization in days, mean (SE) | | 8.3 | 10.4 | 10.2 | 10.1 | 6.7 | 10.5 |
| SE = Standard error | |  |  |  |  |  |  |
| *Obesity includes all body mass index ≥30kg/m^2^  †New life-threatening complications include intubation and death | | |  |  |  |  | |
