# Supplementary material for: Predictors of life-threatening complications in relatively lower-risk patients hospitalized with COVID-19
Source: PLoS One. 2022 Feb 15;17(2):e0263995. doi: 10.1371/journal.pone.0263995 (PMC8846540; doi:10.1371/journal.pone.0263995)
Supplement: Attachment — Submitted filename: Response to Reviewers.docx [file pone.0263995.s003.docx]

Dear Editor,

Thank you for reviewing our manuscript and for your thoughtful feedback. We have addressed the limitations noted within the review, further clarifying our rationale for the inclusion and exclusion criteria used within the study, and have elaborated on context of the study findings. Please find a response to each point raised in the review.

1. *As shown below, I think you need to exclude from the study all those that have been proven to be a risk in at least one meta-analysis. (https://www.cdc.gov/coronavirus/2019-ncov/hcp/clinical-care/underlyingconditions.html)*
   a. *Pregnancy and recent pregnancy*
   b. *Smoking, current and former*

RESPONSE:

With regard to the exclusion criteria for our sample, classification as “relatively lower-risk” was based on the absence of preexisting comorbid conditions, defined using the Charlson Comorbidity Index (CCI), which itself predicts 10-year survival using age and the presence or absence of 16 health conditions, rather than on the CDC list of underlying medical conditions associated with higher risk for severe COVID-19, which has continued to evolve over the course of the pandemic. Smoking and pregnancy are not conditions in the Charlson Index. As a result, relatively-lower risk adults with a history of smoking (n=33) or pregnancy (n=15) were included in our main analytic sample (n=963). The number of pregnant individuals in our sample has been added to *Table 1*.

In *Supplementary Appendix 2* (now S2 Table) of our original manuscript, we reported an additional analysis that expanded our exclusion criteria based on existing literature regarding risk factors for COVID-19 severity and mortality. That analysis excluded patients with hypertension and any severity of obesity (BMI >30mg/k^2^). Based on reviewer comments, the revised manuscript now also excludes patients with a history of smoking or with pregnancy from the relatively low-risk sample in this additional analysis (*S2 Table*). We have clarified this change in the statistical methods section of the revised manuscript (*Page 6*).

2. *The CDC webpage indicates that obesity as a risk for severe COVID-19 illness is a BMI ≧ 30 kg/m^2^. Therefore, in your manuscript, you should describe why you did not adopt this result and defined BMI ≧ 35 kg/m^2^ as obesity.*

RESPONSE:

While prior meta-analyses cited by the CDC have concluded that obesity is associated with the development of critical condition in COVID-19 patients, these studies have noted that the BMI ranges at which the association are observed have varied considerably between individual studies, and have predominantly been observed in ranges over 35 kg/m^2^. (PMID 32686331) Notably, none of the four individual studies included in that meta-analysis showed a statistically significant association between obesity and severe COVID-19 when using a BMI range of 30-35 kg/m^2^ to define obesity. Furthermore, this meta-analysis assessed the relation of obesity to critical condition, rather than the risk of mortality. In the revised manuscript, we have clarified the reasoning for using this selection criteria, stating “we considered patients with severe obesity ([BMI] ≥35 kg/m^2^) to have a preexisting comorbid condition because individual studies have *consistently* shown that severe obesity is independently associated with COVID-19 severity and mortality. Patients with BMI ≥30 but <35 kg/m^2^, referred to as low-risk obesity, were included in our cohort because independent associations with COVID-19 morbidity and mortality have not been consistently established in this group.” (*Page 4*)

3. *COVID-19 illness is a disease in which a high probability of intubation even without complication. However, the intubation rate is low compared to life-threatening complications in this study, and it is questionable whether all these complications were really life-threatening complications. You should show details of how these illnesses were diagnosed in this study.*
   a. *What kind of arrhythmia is included?*
   b. *Were there any false positives or contamination in the blood cultures?*
   c. *How did you confirm venous thrombus embolism?*

RESPONSE:

In regards to outcomes observed in our sample, specifically the rate of life-threatening complications, our primary outcome was a composite score of life-threatening complications that included intubation and death. Consistent with reviewer comments about the high probability of intubation without other complication in COVID-19, intubation and death were in fact the most common complications observed in our sample and contributed to a large proportion of all life-threatening complications observed. In regards to details about how illnesses were diagnosed in this study, outcome data were collected by manual abstraction from the electronic medical records, as noted in our methods. We have provided further details about how these outcomes were abstracted, including the following statement: "Abstraction of life-threatening complications was adjudicated based on physician documentation and/or diagnostic results within the electronic medical records. Major arrhythmias included physician documentation of atrial fibrillation, supraventricular tachycardias, ventricular fibrillation and/or ventricular tachycardia. Positive blood cultures were directly abstracted from microbiology reports. Confirmation of VTE was based on imaging such as a CT-PE or ultrasound." (*Page 5*) Moreover, we cited an additional limitation in the limitations section of our manuscript: “Due to inherent limitations in the chart abstraction process, including a lack of complete clinical context, clinical interpretations of blood culture results were not conducted, and potential contaminants may have been included in the analysis. Notably, the prevalence of this complication was low in our sample (3.2% and 3.8% in those <55 and ≥55 years respectively).” (*Page 14*)

4. *The mortality rate of COVID-19 in this population is higher than the general mortality rate (PMID: 33007452). Therefore, you should explain more details about this cohort, such as indications for hospitalization.*

RESPONSE:

The mortality rate of COVID19 has certainly varied across populations, as is reflected in this interesting reference. However, the mortality rate observed in our paper is the mortality rate in a relatively lower-risk patient population hospitalized with COVID-19. It does not represent the fatality rate, which would include the number of deaths among all those in the entire population that may be potentially infected. The mortality rate in our study approaches those seen among other patients hospitalized with COVID-19 during similar time periods.(PMID 32320003) The mortality rate in our sample was lower than other hospitalized cohorts given the relative health of this cohort (they are by definition relatively lower-risk). Regarding the indication for hospitalization in our study, we have added a statement to our manuscript stating that the decision to hospitalize patients was based on the clinical judgement of the admitting physician at the time of presentation and unaffected by this retrospective study.(*Page 4*)

5. *According to this study, living in areas with limited English proficiency was associated with life-threatening complications. This may be a localized problem, and many readers are unfamiliar with New York City, so they may not share this issue. In order to provide beneficial information, please describe the characteristics of the limited English proficiency in New York City.*

RESPONSE:

We have expanded on the linguistic demographics of New York City within our discussion (Page 14), describing the characteristics of limited English proficiency in New York City.

1. *Reviewer Comment:  We note that you have included the phrase “data not shown” in your manuscript. Unfortunately, this does not meet our data sharing requirements. PLOS does not permit references to inaccessible data. We require that authors provide all relevant data within the paper, Supporting Information files, or in an acceptable, public repository. Please add a citation to support this phrase or upload the data that corresponds with these findings to a stable repository (such as Figshare or Dryad) and provide and URLs, DOIs, or accession numbers that may be used to access these data. Or, if the data are not a core part of the research being presented in your study, we ask that you remove the phrase that refers to these data*

RESPONSE:

The data is not a core part of the research being presented and the phrase has been removed.

Thank you for your consideration of our manuscript.

Sincerely,

Christopher J. Gonzalez, M.D., M.S.
Department of Medicine, Weill Cornell Medical College
338 E 66^th^ Street, New York, NY 10065

cjg7003@med.cornell.edu

646-962-5914
